# Longitudinal Relations Between Views on Aging and Perceived Stress: Evidence for Mutual Associations

**DOI:** 10.1093/geroni/igab046.1110

**Published:** 2021-12-17

**Authors:** Hans-Werner Wahl, Anna Kornadt, Markus Wettstein

**Affiliations:** 1 Heidelberg University, Heidelberg, Baden-Wurttemberg, Germany; 2 University of Luxembourg, Esch-sur-Alzette, Luxembourg; 3 German Centre of Gerontology, Berlin, German Centre of Gerontology, Berlin, Berlin, Germany

## Abstract

We investigated the reciprocal longitudinal relation between perceived stress and three established domains of views on aging (VoA): (1) subjective age; (2) attitude toward own aging [ATOA]; and (3) aging-related cognitions including social loss, physical decline, and continuous growth. We also examined the potentially moderating role of chronological age. Data of the German Ageing Survey, comprising two measurement occasions (2014 and 2017) and a sample of 4,588 individuals aged between 40 and 95 years, were analyzed. Controlling for socio-demographic and health-related indicators, cross-lagged models indicated mutual longitudinal relations between VoA and stress. Whether the pathway from stress to VoA or the opposite pathway was stronger varied depending on the VoA considered. With increasing age, most VoA domains were less strongly associated with subsequent perceived stress. Our findings suggest that less favorable VoA predict higher perceived subsequent stress, but they are also preceded and predicted by higher levels of perceived stress.

